# Retrogasserian trigeminal radiofrequency-thermorhizotmoy for trigeminal neuralgia

**DOI:** 10.1007/s00701-024-06074-2

**Published:** 2024-05-10

**Authors:** A. Brinzeu, M. Sindou

**Affiliations:** 1 University of Lyon, Lyon, France; 2 ELSAN Clinique Breteché, Nantes, France; 3 Neuroscience Research Centre, University of Pharmacy and Medicine Victor Babes Timisoara, Timișoara, Romania; 4 Department of Neurosurgery, University of Pharmacy and Medicine Victor Babes Timisoara, Timișoara, Romania

**Keywords:** Trigeminal neuralgia, Rf-Thermocoagulation, Percutaneous, Hartel’s approach

## Abstract

Based on a personal experience of 4200 surgeries, radiofrequency thermocoagulation is useful lesional treatment for those trigeminal neuralgias (TNs) not amenable to microvascular decompression (idiopathic or secondary TNs). Introduced through the foramen ovale, behind the trigemnial ganglion in the triangular plexus, the needle is navigated by radiology and neurophysiological testing to target the retrogasserian fibers corresponding to the trigger zone. Heating to 55–75 °C can achieve hypoesthesia without anaesthesia dolorosa if properly controlled. Depth of anaesthesia varies dynamically sedation for cannulation and lesioning, and awareness during neurophysiologic navigation. Proper technique ensures long-lasting results in more than 75% of patients.

**Supplementary Information:**

The online version contains supplementary material available at 10.1007/s00701-024-06074-2.

## Introduction

Microvascular decompression (MVD) is considered the first option to treat refractory classical trigeminal neuralgia (TN) [3]. However, when imaging cannot demonstrate neurovascular conflict or in cases of idiopathic or secondary TN lesioning techniques can be helpful. Several options exist from various percutaneous techniques to radiosurgery. Among these we describe the technique for radiofrequency thermo-rhizotomy (RfThR [5, 9]) refined through 4200 procedures. Its goal is achieving hypoesthesia in the trigger zone (TZ) by selectively coagulating the corresponding fibres through navigation in the trigeminal system with radioscopy and neurophysiologic testing accessing this percutaneously through Hartel trajectory [4].

## Relevant surgical anatomy (Fig. 1)

**Fig. 1:**
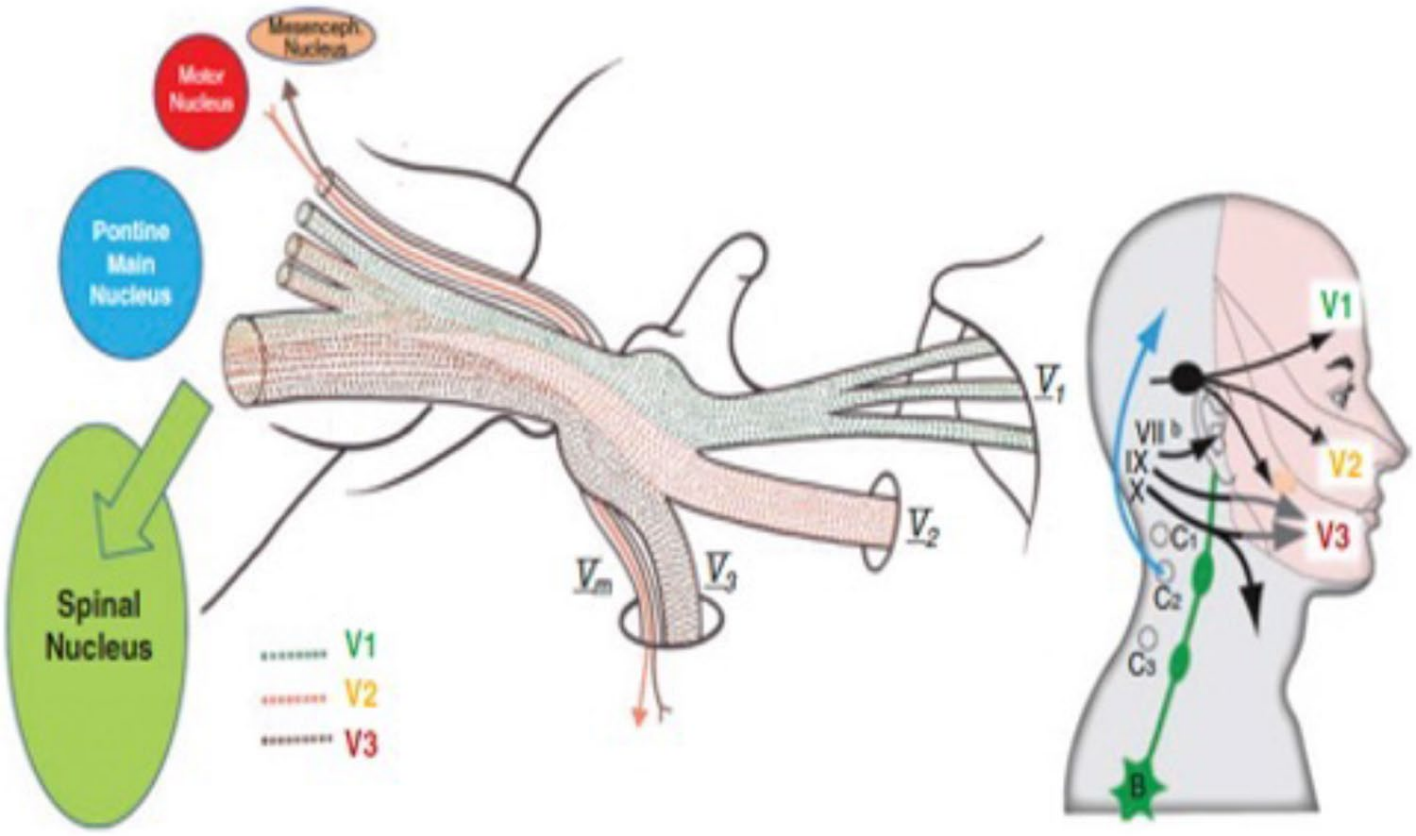
Relevant anatomy of the trigeminal system from the periphery to the brain stem. The three sensory branches of the trigeminal nerve (V1 ophthalmic, V2 maxillary, V3 mandibular) are formed by sensory fibers from the face and cranial mucosae that converge to the sensory trigeminal ganglion. Behind it lies the triangular plexus through which the fibers pass but keep a somatotopic organization. Fibers from the V1 branch remain superomedial while those from the V3 branch are inferolateral. This overall organization is kept through the trigeminal root running within the cerebello-pontine angle cistern. The ganglion and the triangular plexus are in the trigeminal Meckel’s Cave and the plexus is surrounded by CSF (trigeminal cistern). At the trigeminal root entry zone the fibers change their organization from somatotopic to functional to reach their respective nuclei according to their modality: pain fibers towards the spinal trigeminal nucleus, tactile ones towards the main nucleus in the pons, proprioceptive fibers towards the mesencephalic nucleus. In the midpons is situated the motor trigeminal nucleus whose fibers exit the pons via a distinct root(let) exiting above the main root. This will then descend adjacent to V1 to the triangular plexus where it passes below the plexus diagonally from posterior to anterior and medial to lateral. It then joins the V3 peripheral branch exiting adjacent to it through the FO in which it is situated posterior and lateral

The Rf-ThR should be retrogasserian to decrease conduction of the axons of the ganglion cells to the brain stem and not induce retrograde degeneration in the dendrites towards the skin and mucosae. The target should be the portion of the triangular plexus (TP) corresponding to the pain and the trigger zone of the patient [7]; this is situated within the trigeminal Meckels’ Cave (TC), posterior to the semilunar, trigeminal ganglion. The TC is just anterior to the petrous ridge, posteriorly, inferiorly and laterally to the cavernous sinus.

The TP – the ideal target – is somatotopically organised in correspondence to the trigeminal ganglion anteriorly [2]. The plexus is situated on the anterior face of the petrous bone in a oblique, almost horizontal position, V1 fibres are situated medially and superiorly and V3 fibers inferiorly and V2 fibers in between. Vm runs beneath the plexus towards the foramen ovale (FO) where it exits medially and posteriorly to V3. Posterior to the plexus and beyond the upper petrous ridge, the trigeminal root has V1 fibers supero-medially, V3 fibers infero-laterally and V2 fibers in between. Access to TC is through the FO situated antero-lateral to the Cave.

## Description of the technique

### Operative room setup (Fig. 2)

**Fig. 2:**
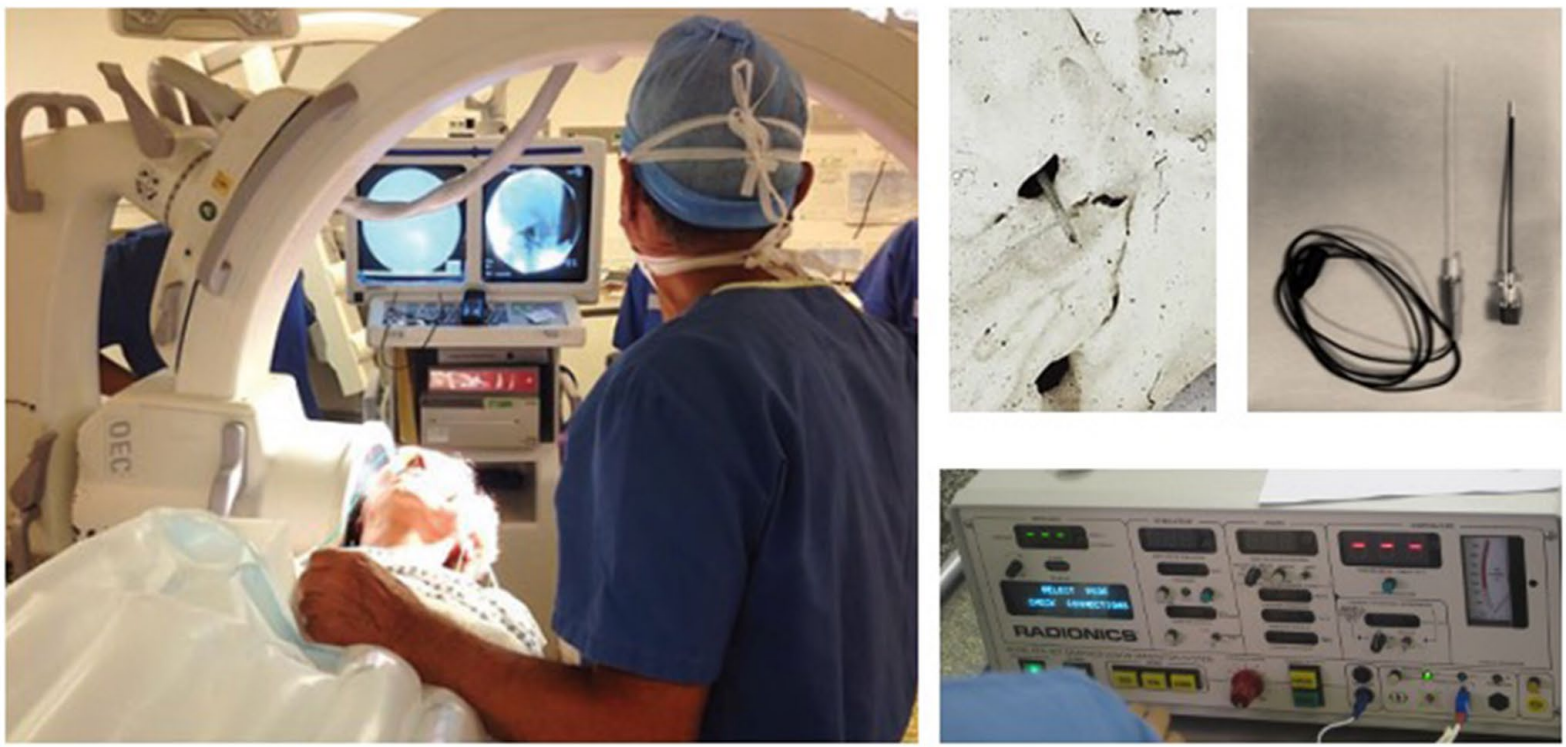
OR Setup for Rf-TR. The patient is supine with the head on a horseshoe head rest to clear the shoulders to place the plate of the C-Arm close to the face on the contralateral side to the pain. Also on this side is placed a unilateral nasal canula delivering oxygen. The C-Arm overarches the patient, and both the screen of the radiology machine and the anesthetist are placed headside. The surgeon directly faces the patient on the side of the pain with the instrument table and the RF machine lateral to him. The RF machine allows for complete control of the current for stimulation. Frequencies of 5 Hz are used to be able to see masticatory and trigemino-facial reflexes. Sensory threshold should be below 400 mV and is carefully controlled. The machine is then switched to radio frequencies to create the thermal lesion. The probe has a thermiresistance, the temperature should be kept below 75 degrees

Patient is supine, the surgeon faces the patient on the side of the pain, the anaesthesiologist headside. The ring of the C-Arm overarches the patient with the detector touching the contralateral temporal region; its screen is cranial directly facing the surgeon. The instrument table and the RF-machine will be just to the side with its neutral electrode on the shoulder for example.

### Anaesthesia

Cannulation of the foramen and lesioning are painful, but good communication is necessary during the navigation phase. Local anaesthesia would be counterproductive hindering sensory testing; sedation of varying depths is therefore used with repeated rapid transition between. This should be at the discretion of the anaesthetist but most often involves a pure total intravenous target-controlled infusion of remifentanyl possibly supplemented with boluses of propofol. Monitoring is standard for sedation.

### Landmarks

**Fig. 3:**
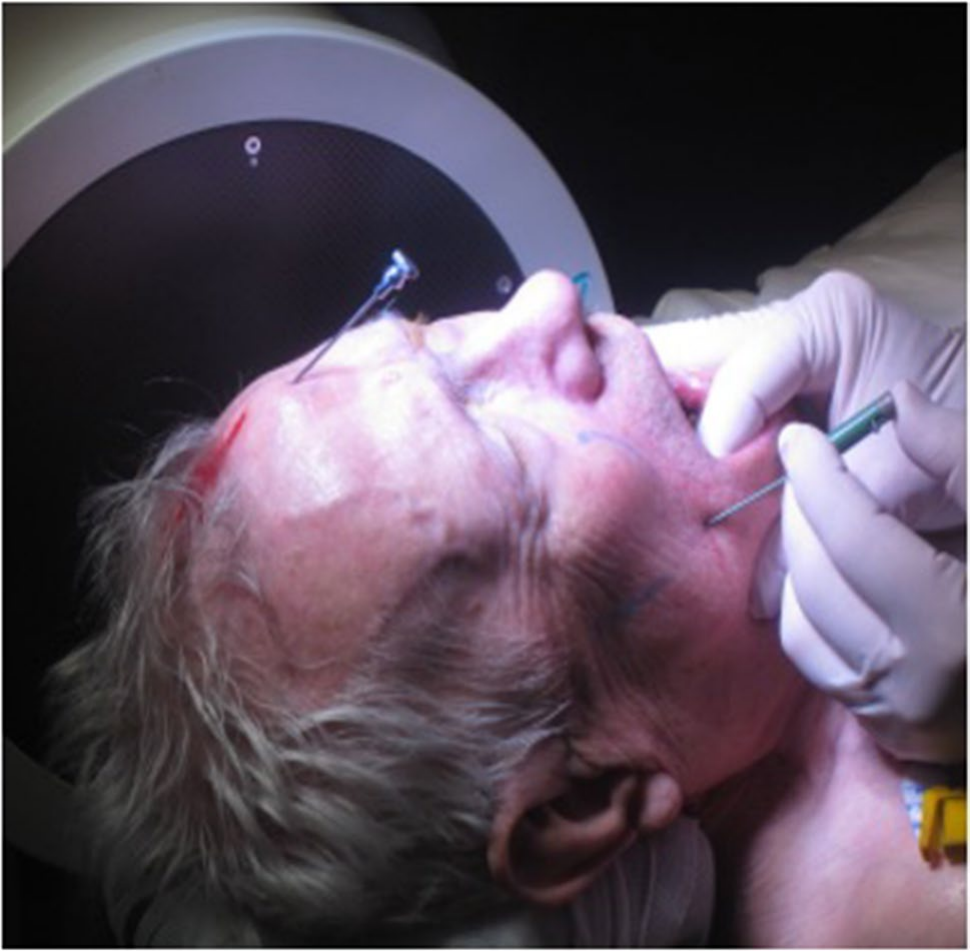
Approach to the Foramen ovale (FO) through Hartel’s trajectory. Landmarks are clearly marked on the face. The entry point is situated 60 mm lateral to the middle of the interlabial line. A point 35 mm anterior to the anterior wall of the external acoustic canal in the orbito-meatal plane, on the inferior zygomatic edge. The pupil in neutral position or a point situated immediately below it on the inferior orbital rim. These three points determine the base of a triangular pyramid with the apex situated at the level of the FO. The needle is inserted through the cheek with the index finger inside the mouth to control the integrity of the buccal mucosa and guide the direction of the needle towards FO through the pterygo-maxillary fossa, and avoiding the lateral pterygoid process

To reach the TC the needle is inserted through the cheek and the pterygo-maxillary fossa lateral to the pterygoid process to the FO. The virtual space created to target the FO has precise facial landmarks demonstrated in Fig. 3 measured and marked on the cheek. These three points designate the base of a triangular pyramid with the apex at the level of FO constituting the trajectory for Hartel’s approach. On its way several anatomical structures of the craniofacial region may be encountered as described in detail by elsewhere [1].

### Needle insertion

**Fig. 4:**
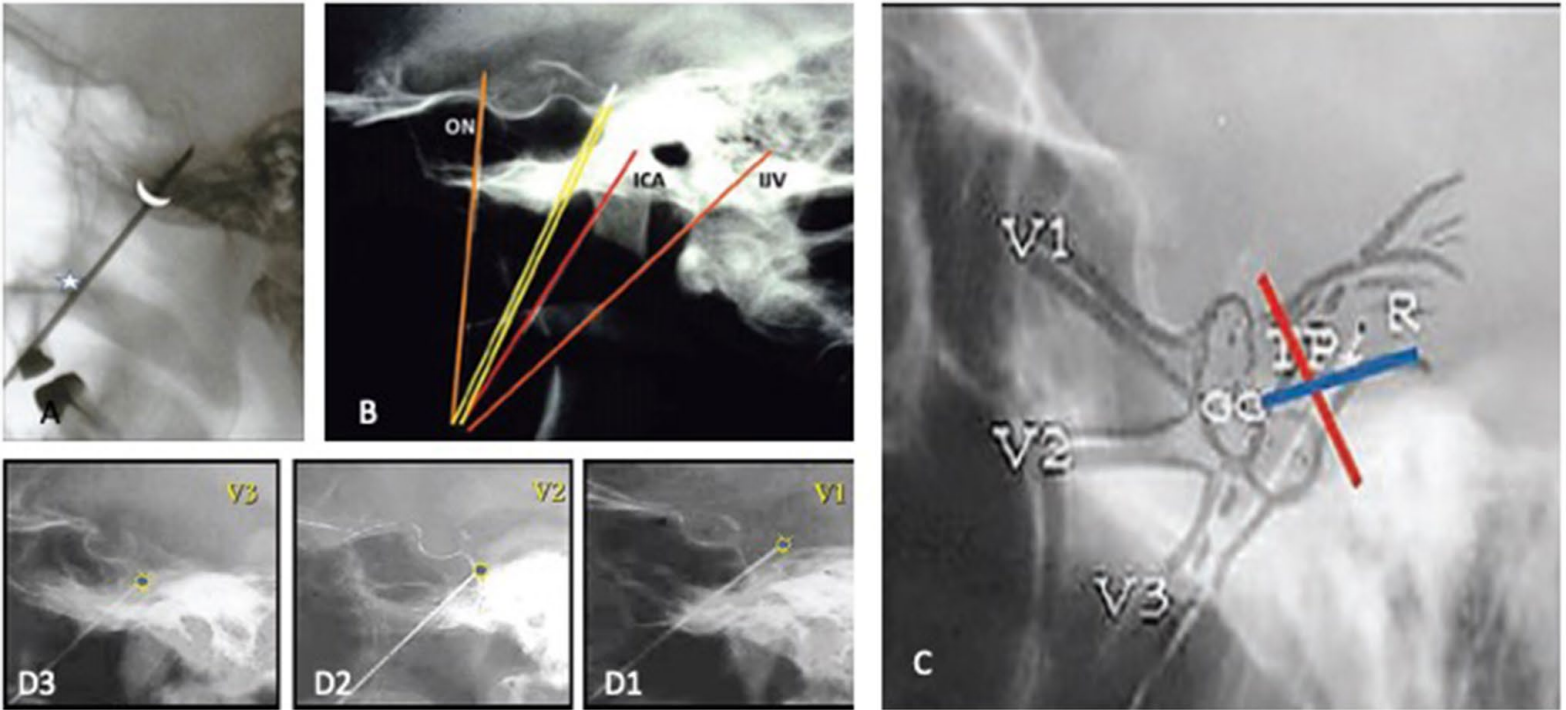
Fluoroscopic control of the position of the needle. Imaging control should be performed at each step of the procedure. **A** strict lateral view is essential (this is checked by aligning the acoustic meati). A The FO is marked by the demilune. An important radiological landmarking the face is the posterior angle of the maxillary sinus (white star). The needle should draw a line between these two points. The tip of the needle should be placed in the triangular plexus. The landmark is the intersection of the clivus and the upper petrous ridge. **B** False trajectories. An excessive postero-lateral direction could puncture the internal jugular vein (IJV) at jugular foramen or the internal carotid artery at entrance into the petrous carotid canal. An excessive medial direction could enter the foramen lacerum and injure the internal carotid artery (ICA) at its C5 segment. An excessive anterior direction could penetrate the orbital apex through the inferior orbital fissure and injure the optic nerve (ON). The appropriate trajectory is yellow. **C** Projection of the peripheral (V1, V2 and V3) branches, ganglion (GG), and trigeminal plexus (TP) on a lateral skull X-Ray. The triangular plexus is situated at the intersection of the projection of the clivus and upper petrous ridge. **D** Respective projections for the probe for V1-V2-V3 lesions

The needle is inserted perpendicular to the skin. The index finger is placed inside the mouth to prevent piercing of the mucosa and palpate its progression. Medially the lateral pterygoid plate may hinder advancement if hypertrophied. The needle is guided towards the FO under fluoroscopic control to avoid wrong trajectories (Fig. 4A, B) [10]. Reaching the FO a small masseter jerk is felt; after the needle is followed on radioscopy passing through the FO, its tip should target the very intersection between the clivus and upper petrous ridge (Fig. 4C). A purely lateral view is essential and this is checked by aligning the acoustic meati (Fig. 4D). Stimulation can begin and therefore the patient must be awakened.

### Navigation in the triangular plexus (Fig. 5)

**Fig. 5:**
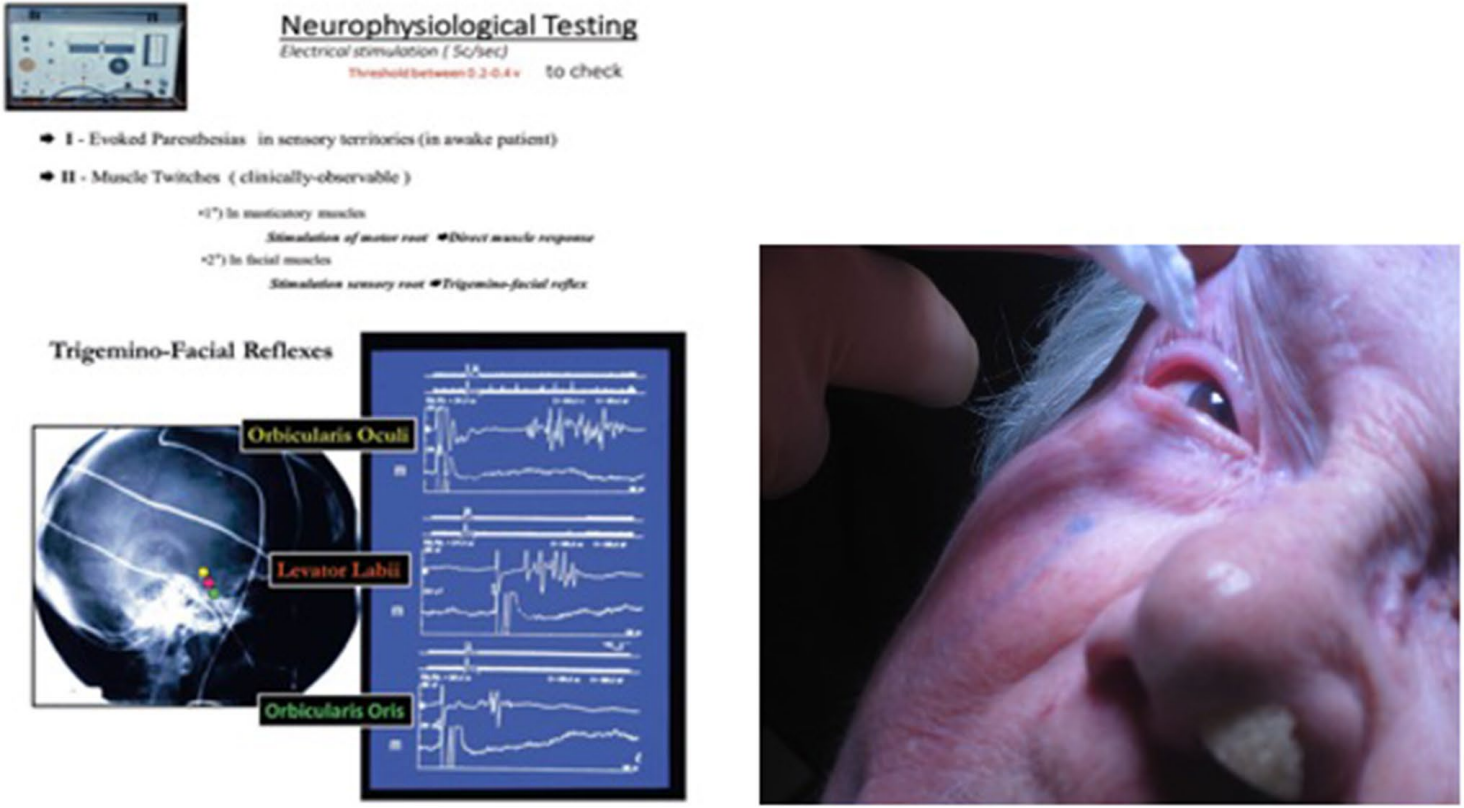
Neurophysiological guidance. Stimulation is used to: 1 Evoke paresthesias in the sensory territory touched by the needle tip, for this a 5 Hz stimulation is used and communication with the patient allows for good sensory targeting. The threshold for sensory stimulation should be between 0.1-0.4 v (100-400 mV). Above 400 mV the needle is too far from the target fibers and should be replaced; 2 Evoke muscle twitches in the masticatory muscles in order to avoid the Vm branch. These masticatory responses are specific for Vm and if evoked at below 400 mV the needle should be replaced; 3 Evoke trigeminofacial reflexes. They have a localizing value concerning the position of the needle within the triangular plexus. Thus a location in V1 will give responses in the orbicularis oculi, V2 in levator labii, V3 in orbicularis oris; 4 The corneal reflex must be tested through the entire procedure to ensure that corneal hypoesthesia is avoided

Sensory stimulation begins at 5 Hz aiming to evoke paraesthesia in the TZ, and the position of the needle is adjusted until this is achieved at intensities below 0.4 V (400 mV).

Masticatory responses at 100 mV signify proximity to Vm requiring uadjustment.

Muscular twitches associated with the sensory stimulation may be observed in the facial muscles. They correspond to trigeminofacial reflexes (TFR) that have demonstrated localizing value [8].

Once the stimulation produces responses in the TZ division territory a test lesion is performed with temperatures in the order of 45–50° (pain threshold) for 20 ± 10 s with the patient still awake. This should evoke mild hypoesthesia in the desired area, otherwise the needle is readjusted. Then depth of anaesthesia is increased before lesioning.

### Lesion creation

A lesion is created by increasing the temperature to 65–75 degrees for sequences of 60 s. This is performed by increments of 5 degrees, and the patient awakened and tested between to verify the hypoesthesia. Corneal reflex is checked each time and if diminished the procedure halted.

### Verification

Hypoesthesia is verified by pinprick at each step and when the TZ is covered, preferably with a significant overextension, the procedure is halted.

### Operative report

includes

- imaging of the needle when lesioning
- stimulation parameters and reactions including TFRs
- temperature and duration of lesions
- the pre and post-lesional sensory observations
- any corneal hypoesthesia

### Indications

Following, the general indications for percutaneous and SRS procedures for TN:

- Rf-TR is the first line surgical treatment in MS related TN.
- Idiopathic TN – patients with primary TN not harbouring a clear cut NVC
- Trigeminal neuralgias in fragile patients
- Recurrence after MVD properly done
- Some neoplastic TN

## How to avoid complications

Complications related to the trajectory are avoided by precise landmarks and radiologic verification thus reducing: hemosialorhea (by piercing the parotid duct), pterygo-maxillary haematoma (injury to the maxillary artery), obstruction of the Eustachian tube and (rarely) carotid puncture – potentially dramatic.

Sequelae whether severe (anaesthesia dolorosa, corneal hypoesthesia with keratitis) or lighter (bothersome facial numbness or masticatory weakness) are avoided through proper testing and communication with the patient.

A too high tip of the probe in the CPA cistern risks of trochlear nerve palsy, whereas a too low tip. (inside the gasserian ganglion) risks trophic peripheral ulcers.

## Specific perioperative considerations

Preparation

- Painful territory, TZ and prior hypoesthesia.
- Imaging: en-route pathology or vascular anomalies.
- Consider navigation or O-Arm/intraoperative-CT if difficulties in cannulating FO (platybasia).

Patient

- Explain: gain trust and collaboration.
- Communicate:
  - Objective: obtaining hypoesthesia of the TZ
  - Testing procedure: sensory stimulation and testing
- Verify: no anticoagulants/antiplatlets

Anaesthesia

- The painful steps—heavy sedation, for:
  - puncture and cannulation of FO
  - lesioning
- Cooperation during testing (light sedation)

Postoperative

- Artificial tears and ocular vitamin A
- Reduce antiepileptics only by half immediately, adjusted thereafter.
- Ophthalmology evaluation R/O corneal hypoesthesia or keratitis.

## Informed consent

The one in one thousand (1/1000) vital risk is mentioned.

Significant numbness must be announced.

Corneal lesions and recurrence are the key points.

## Conclusion

**Fig. 6:**
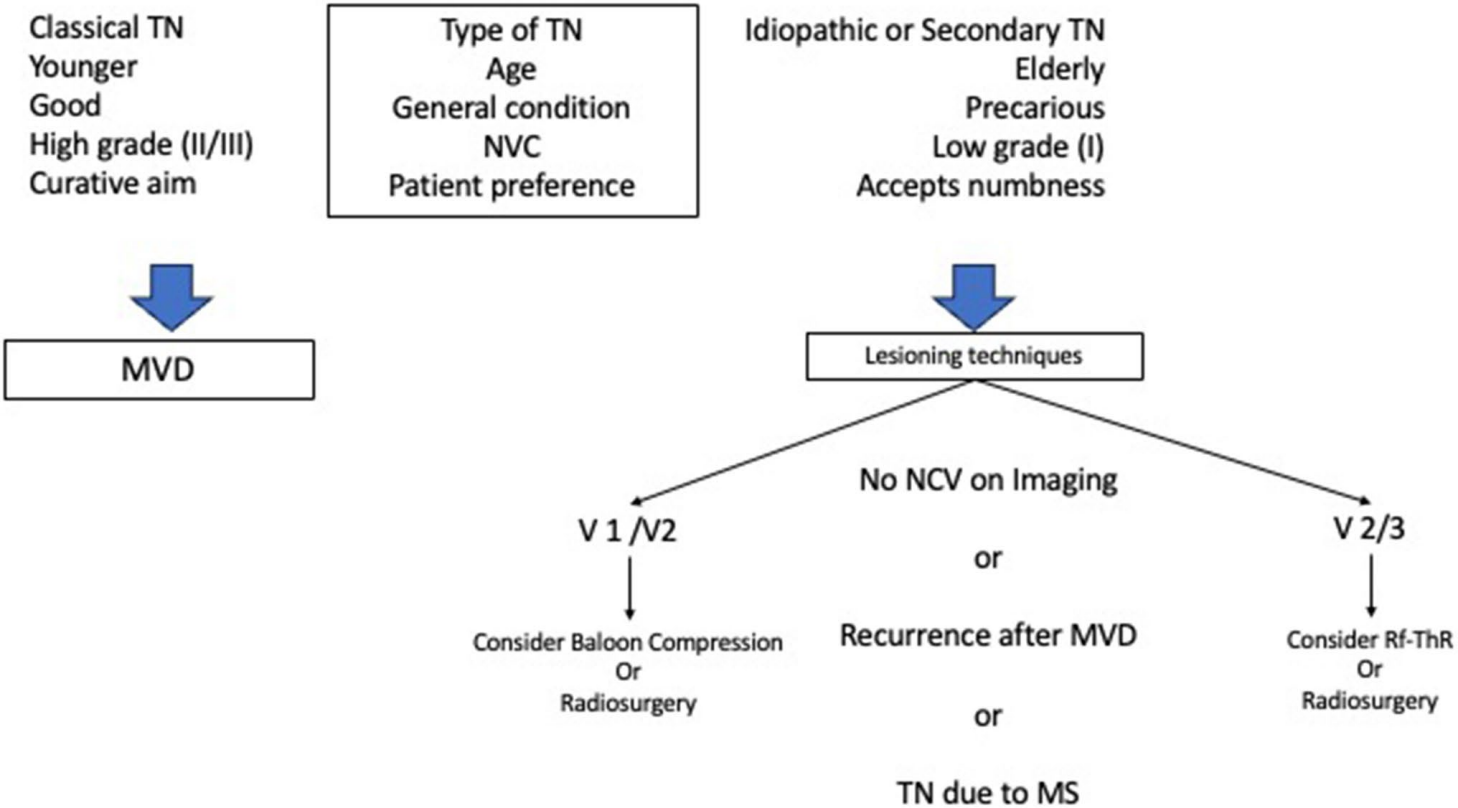
Decision algorithm for surgical treatment of TN. Classical means by definition with a significant probability that the TN is due to an NVC [3]

Rf-ThR is a key element in armamentarium of TN treatment (Fig. 6).

## Supplementary Information

Below is the link to the electronic supplementary material.

Supplementary file1 (MP4 246 MB)

## Data Availability

The authors declare all original data is available for review representing partial patient files as the case may apply.

## Code availability

Not aplicable.
